# Supplementary material for: TALPID3 and ANKRD26 selectively orchestrate FBF1 localization and cilia gating
Source: Nat Commun. 2020 May 4;11:2196. doi: 10.1038/s41467-020-16042-w (PMC7198521; doi:10.1038/s41467-020-16042-w)
Supplement: Supplementary file 1 — Supplementary Information [file 41467_2020_16042_MOESM1_ESM.pdf]

Supplementary Information

TALPID3 and ANKRD26 selectively orchestrate FBF1 localization and cilia gating

Yan *et al.*

The file contains:

1. Supplementary Figures 1-10.
2. Supplementary Table 1 and 2.

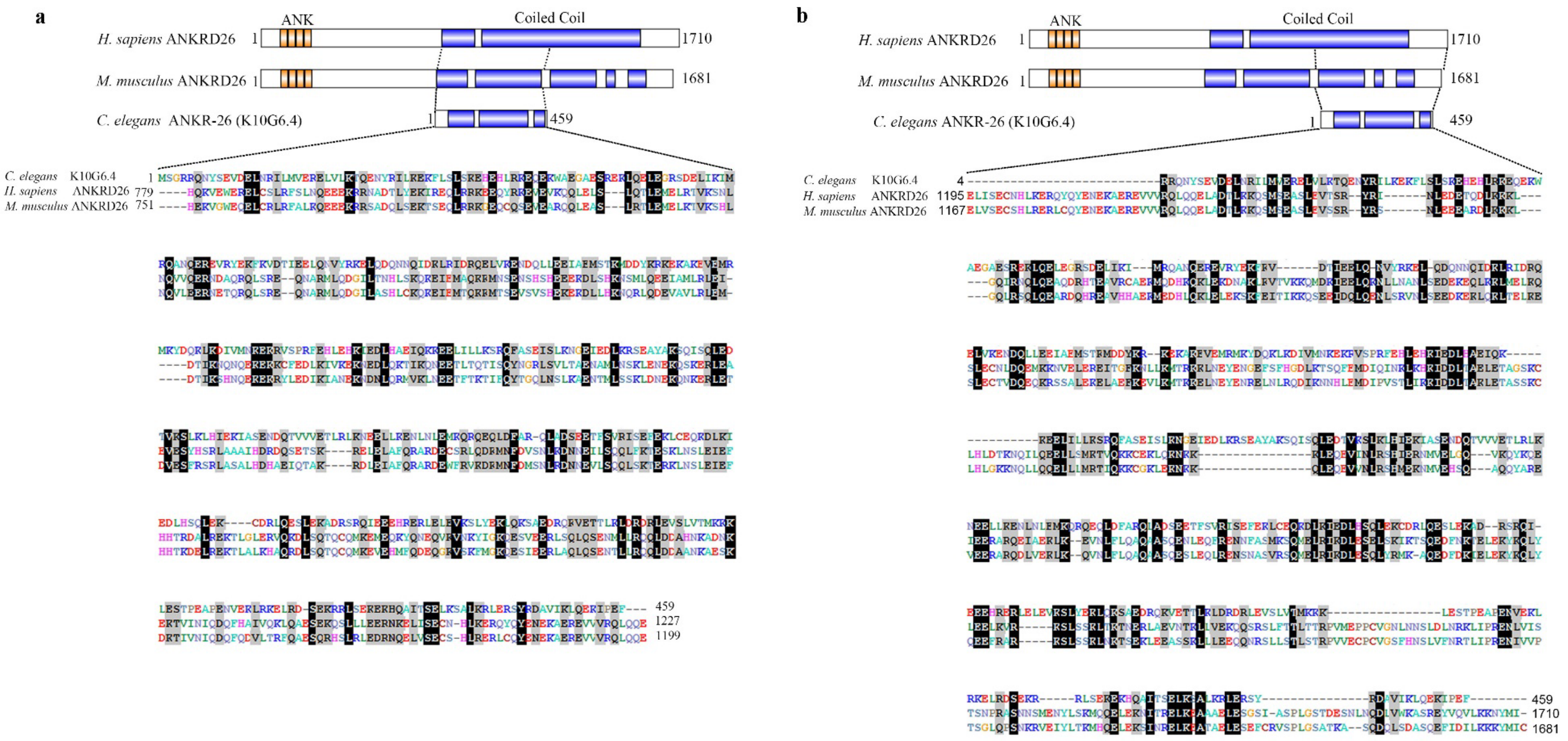

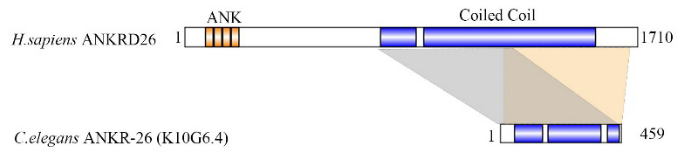

C .elegans	Human	Identity(%)	Similarity(%)
ANKR-26(FL:1-459aa)	ANKRD26(779-1227aa)	17.52	39.32
ANKR-26(FL:1-459aa)	ANKRD26(1222-1710aa)	19.04	38.65

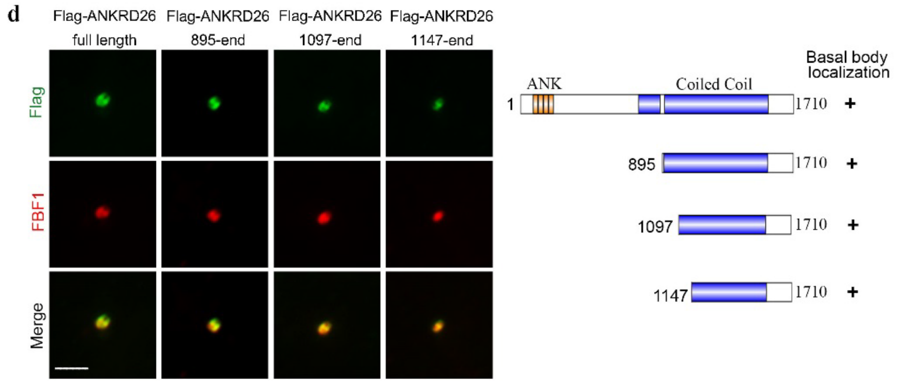

Supplementary Figure 1. *C. elegans* ANKR-26 is homologous to the C-terminus of human ANKRD26.

a. Sequence homology between *C. elegans* ANKR-26, aa 779–1227 of human ANKRD26 and aa 751–1199 of mouse ANKRD26. **b.** Sequence homology between *C. elegans* ANKR-26, aa 1222 to the C-terminal end of human ANKRD26 and aa 1194 to the C-terminal end of mouse ANKRD26. **c**. Schematic of human ANKRD26 and *C. elegans* ANKR-26. *C. elegans* ANKR-26 is homologous to the C-terminal coiled-coil domain of human ANKRD26. Coiled-coil sequences are likely to be duplicated during evolution. **d.** The C-terminal coiled-coil domain is sufficient to target ANKRD26 to the centrosome. Bar, 2μm.

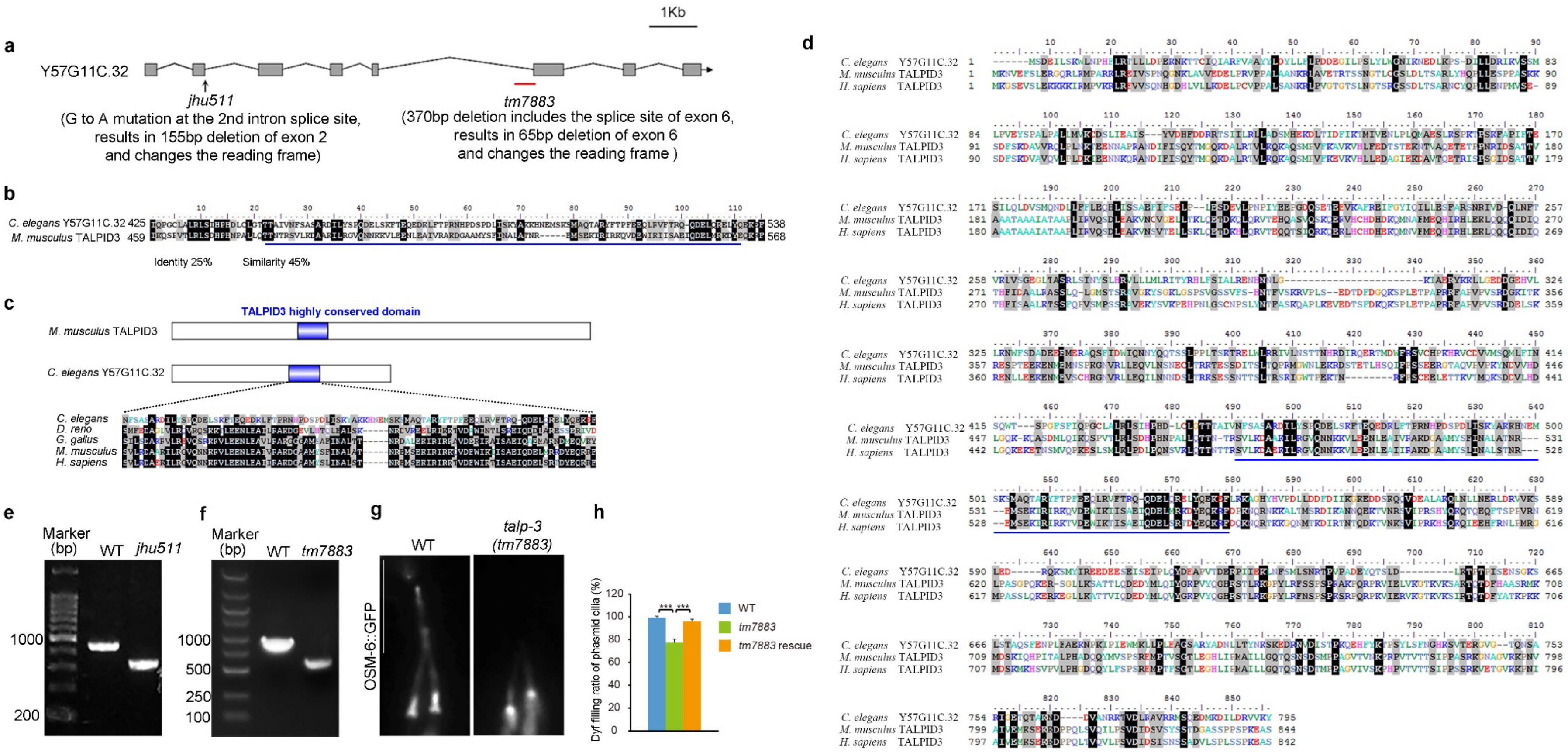

Supplementary Figure 2. Isolation and identification of *talp-3* mutants in *C. elegans*.

a. Genomic structure of *C. elegans Y57G11C.32* (*talp-3*). A G-to-A mutation occurs at the 2nd intron splice site in *jhu511* mutants. This mutation leads to the loss of 155 bp in exon 2 and a reading frameshift. *tm7883* was obtained from the National Bioresource Project (NBRP) in Japan. A total of 370 bp are deleted in the *tm7883* mutant. This deletion changes the acceptor site of the 5th intron and leads to the loss of 65 bp of exon 6, resulting in a reading frameshift. **b.** Sequence homology at aa 425–538 of Y57G11C.32 and aa 459–568 of mouse TALPID3. The highly conserved TALPID3 region is underlined in blue. **c.** Schematic of mouse TALPID3 and *C. elegans* TALP-3 and multiple sequence alignment of highly conserved regions of TALPID3 from vertebrates and *C. elegans*. **d.** Multiple sequence alignment of Y57G11C.32 protein sequence with human and mouse TALPID3 proteins. **e.** PCR analysis of a cDNA product spanning the mutation site in *talp-3*(*jhu511*). **f.** PCR analysis of a DNA product spanning the mutation site in *talp-3*(*tm7883*). **g.** Images of phasmid cilia labeled with OSM-6::GFP in WT worms and *talp-3(tm7883)*. Bar, 5 µm. **h.** Quantification of the dye-filling ratio in phasmid cilia in WT worms and *talp-3(tm7883)*. Similar to *talp-3(jhu511)*, *talp-3(tm7883)* has partially truncated cilia and is partially defective in dye filling. n=300 worms over 3 independent experiments. Data are represented as the mean value ± s.d. Significant differences were identified by two-tailed unpaired student's *t*-test. No adjustments were made for multiple comparisons. ***, P<0.001. Source data are provided as a Source Data file.

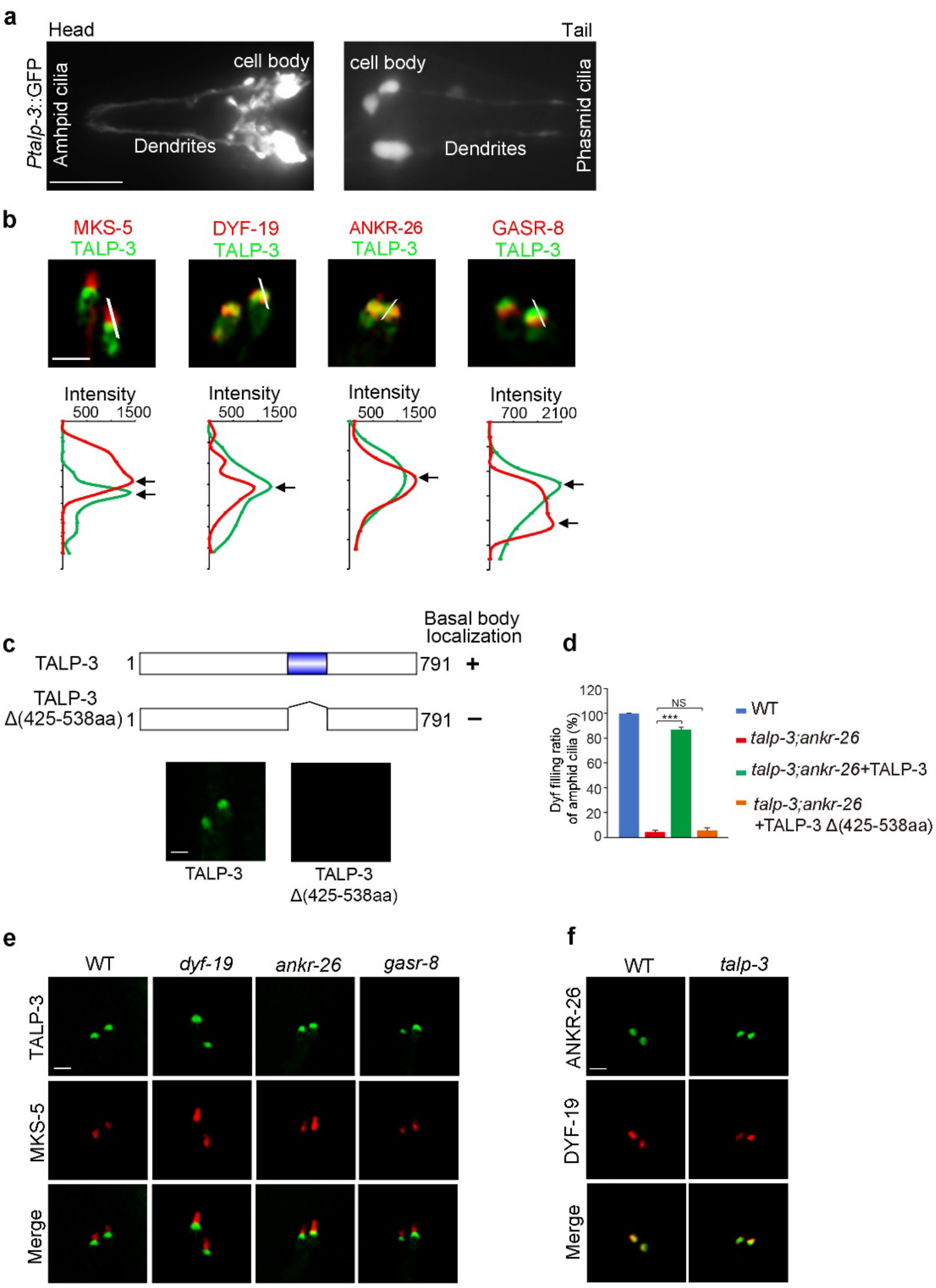

Supplementary Figure 3. **The subcellular localization of TALP-3 in *C. elegans*.**

a. TALP-3 is expressed exclusively in ciliated cells in *C. elegans*. A GFP reporter gene driven by the *talp-3* promoter is expressed exclusively in ciliated cells. **b.** Co-localized images of TALP-3 with TZ or basal body proteins. Corresponding line-scan profiles of fluorescence intensity are shown in bottom panels. **c.** Schematics of full-length TALP-3 and truncated TALP-3 mutants and their localizations at the basal body. **d.** The highly conserved region is required for the basal body localization and function of TALP-3. n=300 worms over 3 independent experiments. Data are represented as the mean value ± s.d. Significant differences were identified by two-tailed unpaired student's *t*-test. ***, P<0.001. **e.** DYF-19, ANKR-26 and GASR-8 are not required for the basal body localization of TALP-3. **f.** TALP-3 is not required for DYF-19 and ANKR-26 localization. Bars, 20 µm in (**a**); 1 µm in (**b**, **c**, **e**, **f**). Source data are provided as a Source Data file.

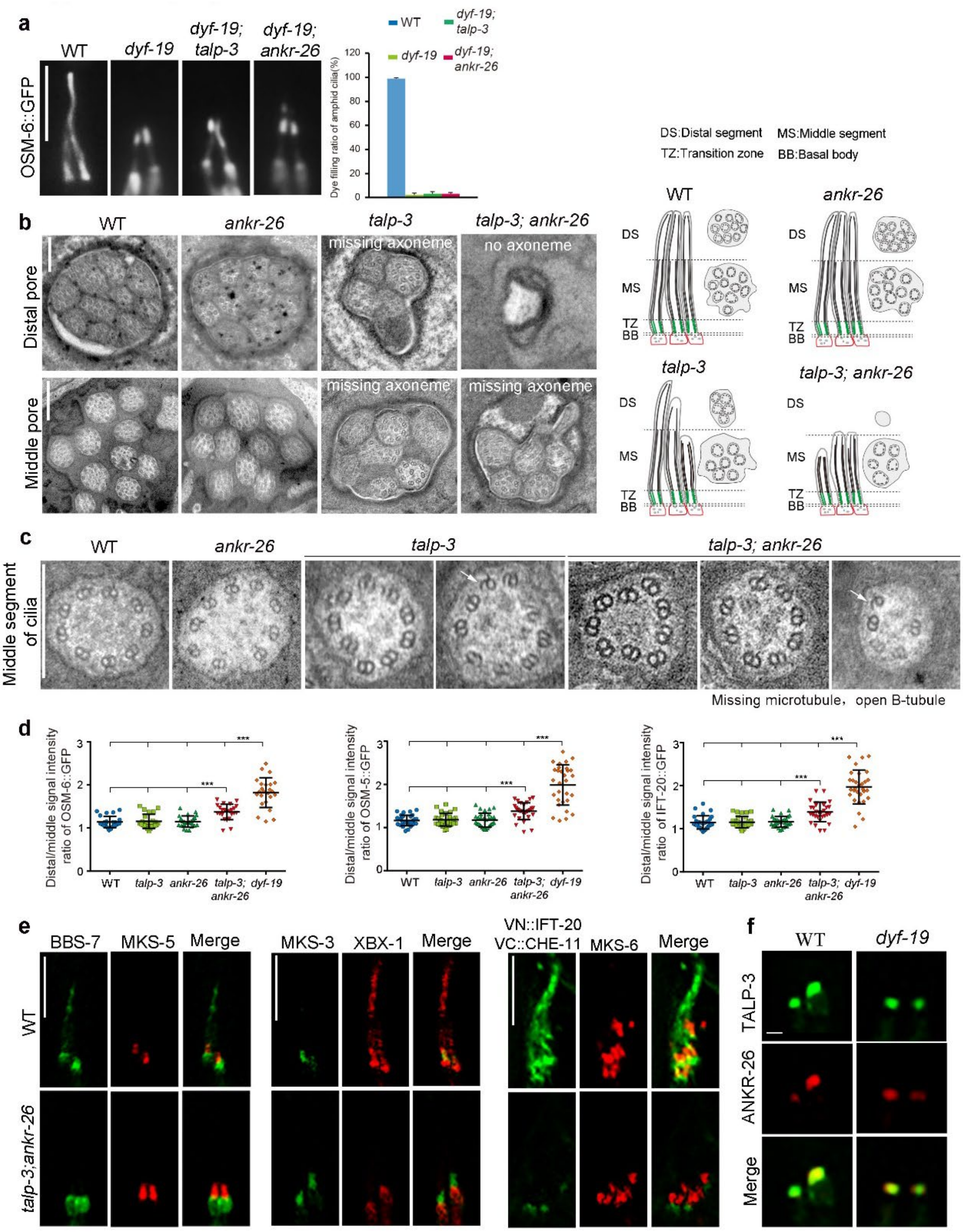

Supplementary Figure 4. Genetic interactions among TALP-3, ANKR-26 and DYF-19.

a. Representative images (left panel) and quantification of the dye-filling ratio of amphid cilia (right panel) in WT, *dyf-19* single-mutant, and *dyf-19; talp-3* and *dyf-19; ankr-26* double-mutant worms. Both the *dyf-19; talp-3* and *dyf-19; ankr-26* double mutants mimic *dyf-19* single mutants, and the abnormal phenotype was not enhanced. n=300 worms over 3 independent experiments. **b.** Transmission electron microscopy (TEM) images of cross-sections of the amphid channel cilia in worms in the indicated genetic background. In WT worms, each amphid channel pore contains 10 cilia. Bars, 200 nm. **c.** TEM images of middle segment of cilia in the indicated genetic background. Missing microtubules and open B-tubules (white arrows) were observed in *talp-3; ankr-26* double mutants. Bar, 200 nm. **d.** Ratio between the distal/middle signal intensity of OSM-6::GFP, OSM-5::GFP and IFT-20::GFP in WT worms, *talp-3* mutant worms, *ankr-26* mutant worms and *talp-3; ankr-26* double mutants. n=24 for OSM-6::GFP, n=30 for OSM-5::GFP and IFT-20::GFP. Each data point represents a single measurement. **e.** Compromised ciliary entry of IFT proteins in *talp-3; ankr-26* double mutants. Compared with their localization in WT worms, the IFT proteins BBS-7 and XBX-1 and the IFT complex VN::IFT-20; VC::CHE-11 were restricted below the TZ in *talp-3; ankr-26* double mutants. Bars, 5 µm. **f.** DYF-19 is not required for TALP-3 and ANKR-26 localization. Bars, 1 µm. All data are represented as the mean value ± s.d. Significant differences were identified by two-tailed unpaired student's *t*-test. No adjustments were made for multiple comparisons. ***, P<0.001. Source data are provided as a Source Data file.

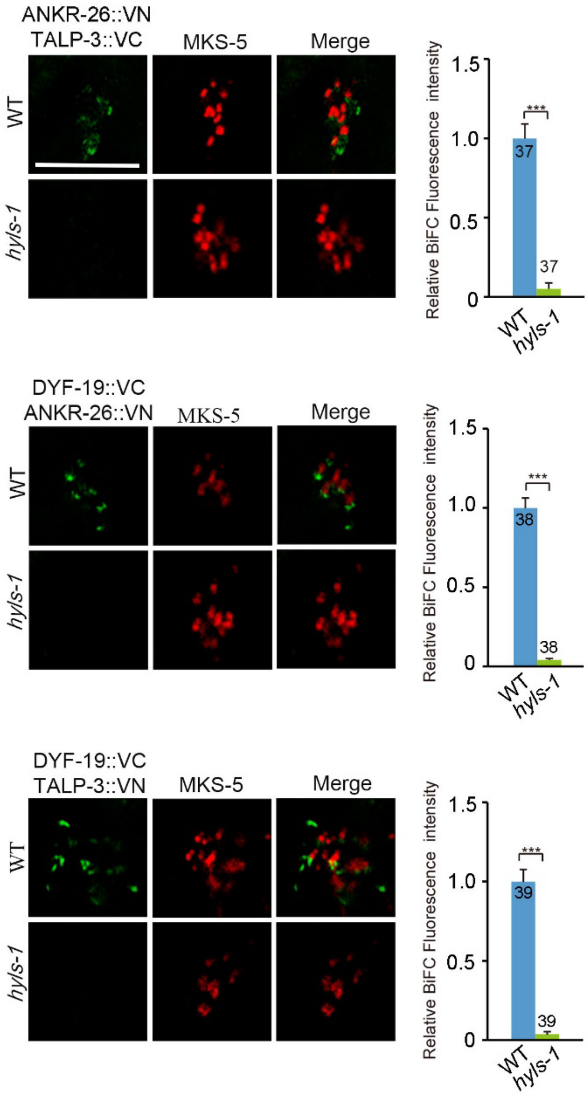

Supplementary Figure 5. BiFC signals between TALP-3 and ANKR-26, TALP-3 and DYF-19, and DYF-19 and ANKR-26 are not observed in *hyls-1* mutants. The n number is indicated in the bars. All data are represented as the mean value ± s.d. Significant differences were identified by two-tailed unpaired student's *t*-test. No adjustments were made for multiple comparisons. ***, P<0.001. Source data are provided as a Source Data file.

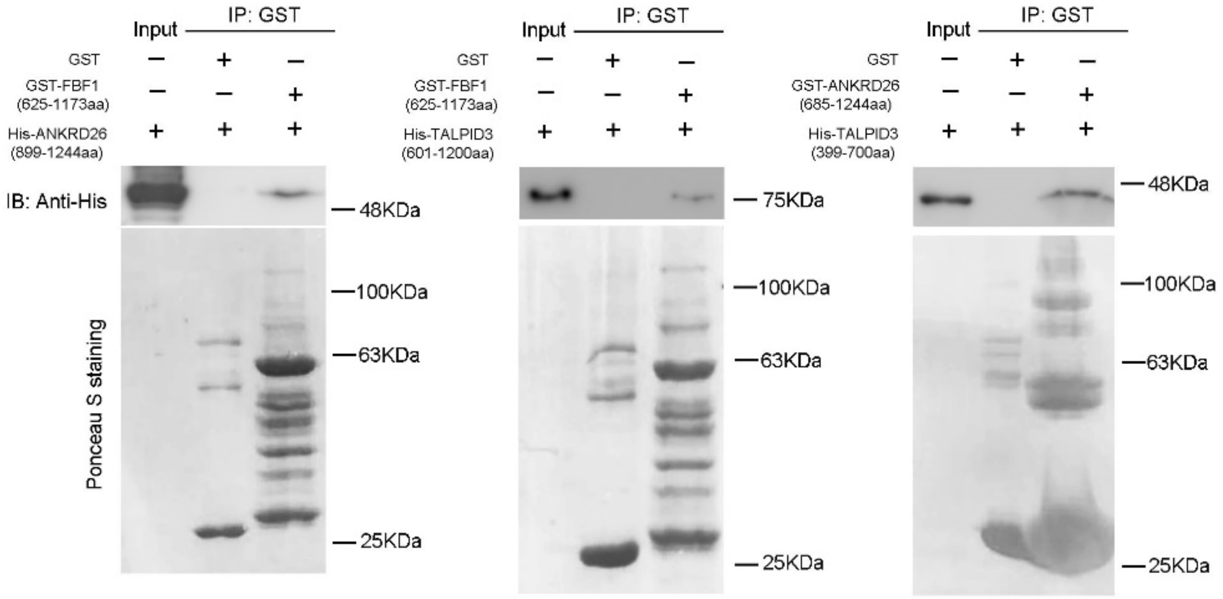

Supplementary Figure 6. Mammalian FBF1, ANKRD26 and TALPID3 interact with each other, as shown by GST pull-down assay.

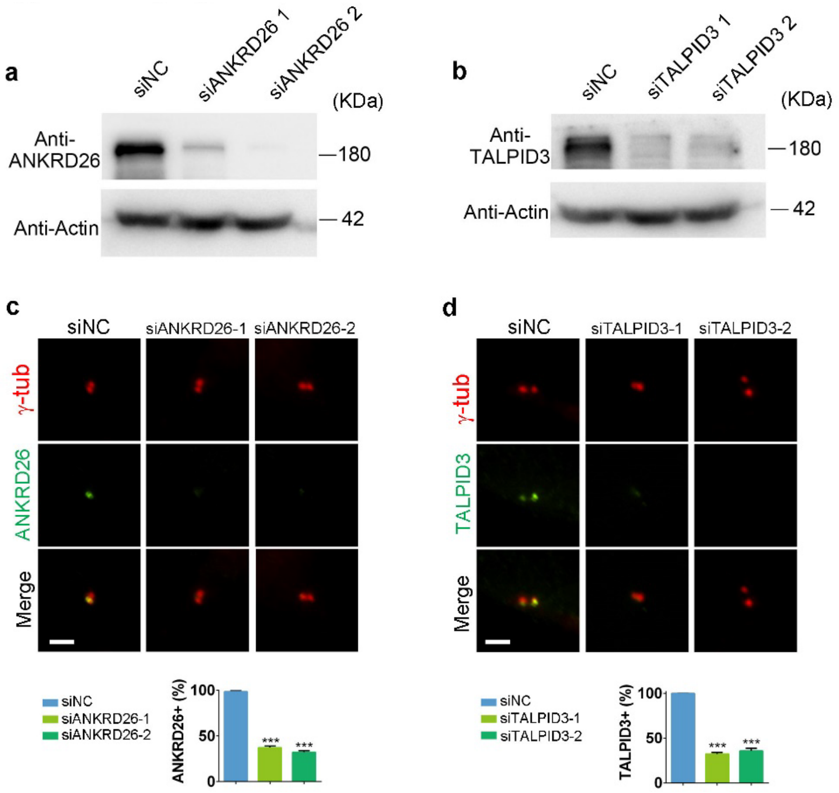

Supplementary Figure 7. Efficiency of siANKRD26- and siTALPID3-mediated knockdown.
RPE cells were treated with negative control (siNC), ANKRD26-specific (siANKRD26 1 and siANKRD26 2) (**a, c**) or TALPID3-specific (siTALPID3 1 and siTALPID3 2) siRNAs (**b, d**) for 48 h. (**a, b**) Cell lysates were collected and analyzed using an immunoblotting assay with specific antibodies. (**c, d**) Upper panel: localization of ANKRD26 and TALPID3 at the basal body was examined by IF microscopy with the indicated antibodies. Lower panel: percentages of cells exhibiting ANKRD26 or TALPID3 localization at the basal body were quantified in a minimum of 100 cells in each group. Scale bars, 2 μm. ***, $P < 0.001$. n=300 cells over 3 independent experiments. All data are represented as the mean value ± s.d. Significant differences were identified by two-tailed unpaired student's *t*-test. No adjustments were made for multiple comparisons. ***, P<0.001. Source data are provided as a Source Data file.

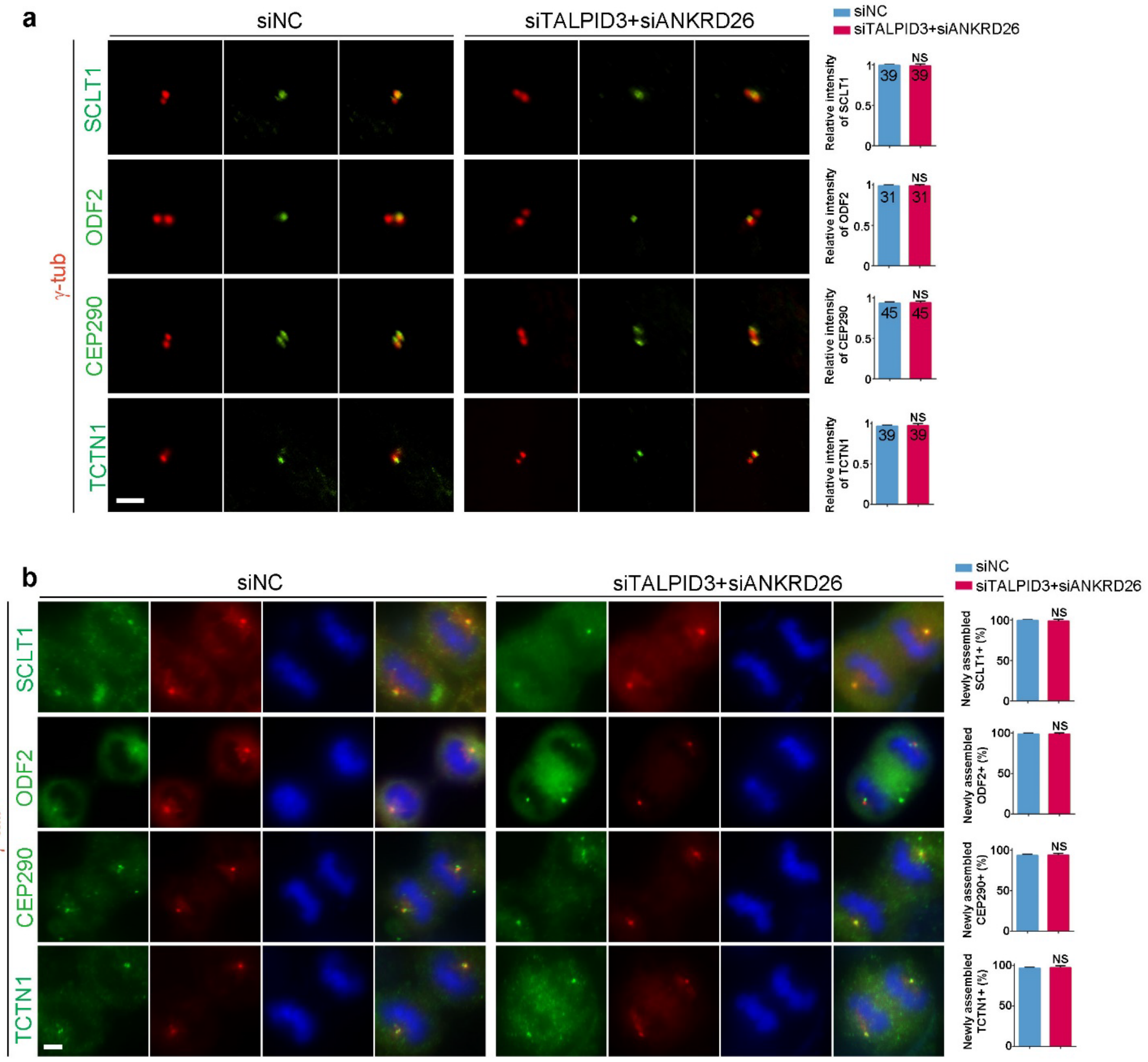

Supplementary Figure 8. Co-depletion of TALPID3 and ANKRD26 does not affect the localizations of SCLT1, ODF2, CEP290 and TCTN1.

a. Localizations of SCLT1, ODF2, CEP290 and TCTN1 in quiescent cells were not affected by co-depletion of ANKRD26 and TALPID3. The n number is indicated in the bars. **b.** Recruitment of SCLT1, ODF2, CEP290 and TCTN1 was normal during centriole maturation upon the co-depletion of ANKRD26 and TALPID3. n=300 cells over 3 independent experiments. Scale bars, 2 µm. All data are presented as the mean ± s.e.m. Significant differences were identified by two-tailed unpaired Student's *t*-test. NS, $P>0.05$. Source data are provided as a Source Data file.

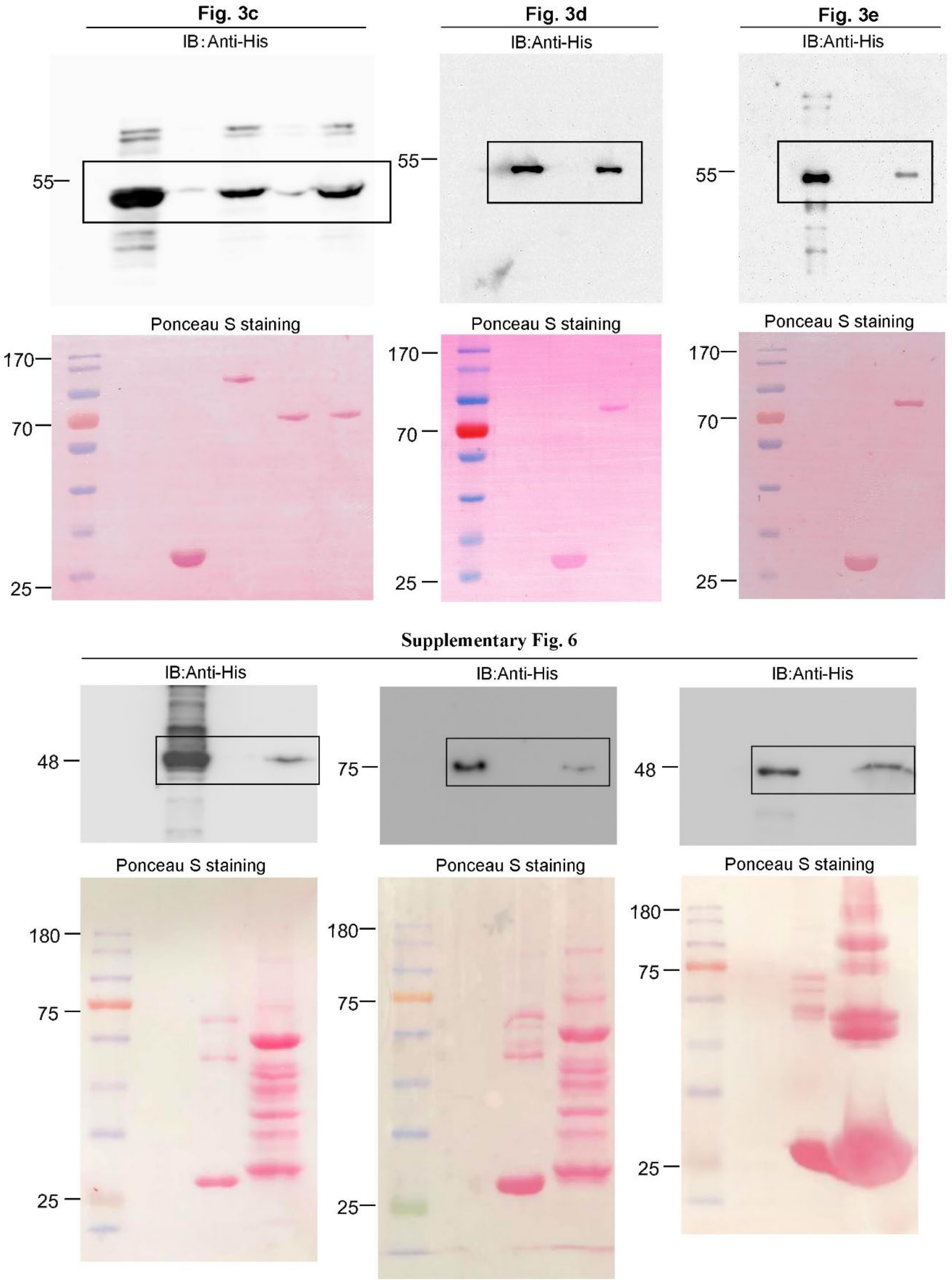

Supplementary Figure 9. Scans of the full western blots in Figure 3 and Supplementary Figure 6.

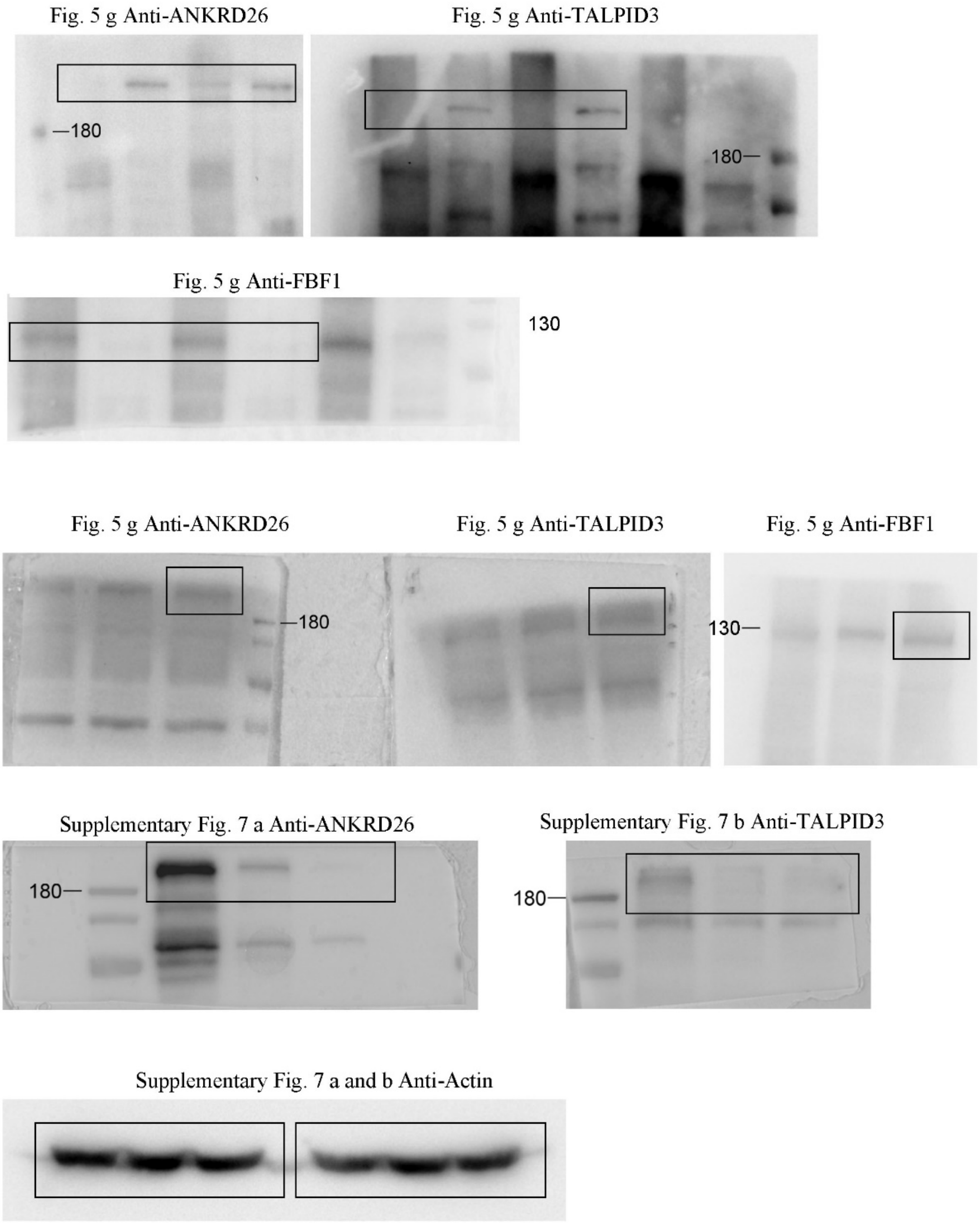

Supplementary Figure 10. Scans of the full western blot in Figure 5 and Supplementary Figure 7.

Supplementary Table 1. Worm strains used in this study.

Strain name	Genotype
QWL58	*talp-3 (jhu511) IV; IsOSM-6*
IsOSM-6	*myIs17 [OSM-6::GFP]*
QWL173	*talp-3 (tm7883) IV; IsOSM-6*
QWL176	*talp-3(jhu511) IV; EX [Parl-13::TALP-3::GFP;+pRF4]*
QWL83	*Ex [Parl-13::TALP-3::GFP+Parl-13::MKS-5::mCherry+pRF4]*
QWL20	*Ex [Parl-13::TALP-3::GFP+Parl-13::DFY-19::mCherry+pRF4]*
QWL284	*Ex [Parl-13::TALP-3::GFP+Parl-13::ANKR-26::mCherry+pRF4]*
QWL302	*Ex [Parl-13::TALP-3::GFP+Parl-13::GASR-8::mCherry+pRF4]*
OD192	*hyls-1 (tm3067) V*
VC2343	*gasr-8 (gk1232) V*
VC1268	*ankr-26 (gk567) II*
QWL164	*hyls-1(tm3067) V; EX [Parl-13::TALP-3::GFP+Parl-13::MKS-5::mCherry+pRF4]*
QWL280	*dyf-19 (jhu455) V; EX [Parl-13::TALP-3::GFP+Parl-13::MKS-5::mCherry+pRF4]*
QWL240	*ankr-26 (gk567) II; EX [Parl-13::TALP-3::GFP+Parl-13::MKS-5::mCherry+pRF4]*
QWL241	*gasr-8 (gk1232) V; EX [Parl-13::TALP-3::GFP+Parl-13::MKS-5::mCherry+pRF4]*
QWL88	*EX [Ptalp-3::GFP+pRF4]*
QWL215	*ankr-26 (gk567) II; IsOSM-6*
QWL213	*talp-3 (jhu511) IV; ankr-26 (gk567) II; IsOSM-6*
QWL57	*Ex [OSM-5::GFP+pRF4]*
QWL13	*Ex [IFT-20::GFP+pRF4]*
QWL11	*Ex [CHE-11::GFP+pRF4]*
QWL198	*Ex [OSM-3::GFP+pRF4]*
QWL55	*Ex [KAP-1::GFP+pRF4]*
QWL62	*Ex [MKS-3::GFP+XBX-1::tdTomato+pRF4]*
QWL19	*Ex [BBS-7::GFP+MKS-5::mCherry+pRF4]*
QWL325	*talp-3 (jhu511) IV; Ex [OSM-5::GFP+pRF4]*
QWL326	*talp-3 (jhu511) IV; Ex [IFT-20::GFP+pRF4]*

QWL223	*talp-3 (jhu511) IV; Ex [CHE-11::GFP+pRF4]*
QWL138	*talp-3 (jhu511) IV; Ex [OSM-3::GFP+pRF4]*
QWL207	*talp-3 (jhu511) IV; Ex [KAP-1::GFP+pRF4]*
QWL188	*talp-3 (jhu511) IV; Ex [XBX-1::GFP+pRF4]*
QWL281	*talp-3 (jhu511) IV; Ex [BBS-7::GFP+pRF4]*
QWL277	*ankr-26 (gk567) II; Ex [OSM-5::GFP+pRF4]*
QWL139	*ankr-26 (gk567) II; Ex [IFT-20::GFP+pRF4]*
QWL278	*ankr-26 (gk567) II; Ex [CHE-11::GFP+pRF4]*
QWL142	*ankr-26 (gk567) II; Ex [OSM-3::GFP+pRF4]*
QWL267	*ankr-26 (gk567) II; Ex [KAP-1::GFP+pRF4]*
QWL220	*ankr-26 (gk567) II; Ex [XBX-1::GFP+pRF4]*
QWL86	*ankr-26 (gk567) II; Ex [BBS-7::GFP+pRF4]*
QWL174	*talp-3 (jhu511) IV; ankr-26 (gk567) II; Ex [OSM-5::GFP+pRF4]*
QWL185	*talp-3 (jhu511) IV; ankr-26 (gk567) II; Ex [IFT-20::GFP+pRF4]*
QWL183	*talp-3 (jhu511) IV; ankr-26 (gk567) II; Ex [CHE-11::GFP+pRF4]*
QWL201	*talp-3 (jhu511) IV; ankr-26 (gk567) II; Ex [OSM-3::GFP+pRF4]*
QWL191	*talp-3 (jhu511) IV; ankr-26 (gk567) II; Ex [KAP-1::GFP+pRF4]*
QWL175	*talp-3 (jhu511) IV; ankr-26 (gk567) II; Ex [XBX-1::GFP+pRF4]*
QWL193	*talp-3 (jhu511) IV; ankr-26 (gk567) II; Ex [BBS-7::GFP+pRF4]*
QWL405	*Ex [OSM-9::GFP+MKS-5::mCherry]*
QWL406	*Is [PKD-2::GFP; CC::GFP];EX[MKS-5::mCherry+pRF4]*
QWL407	*kyIs53 [odr-10::GFP]; EX [MKS-5::mCherry+pRF4]*
QWL408	*talp-3 (jhu511) IV; Ex [OSM-9::GFP+MKS-5::mCherry]*
QWL409	*talp-3 (jhu511) IV; Is [PKD-2::GFP; CC::GFP]; EX[MKS-5::mCherry+pRF4]*
QWL410	*talp-3 (jhu511) IV; kyIs53 [odr-10::GFP]; EX [MKS-5::mCherry+pRF4]*
QWL411	*ankr-26 (gk567) II; Ex [OSM-9::GFP+MKS-5::mCherry]*
QWL412	*ankr-26 (gk567) II; Is [PKD-2::GFP; CC::GFP];EX[MKS-5::mCherry+pRF4]*
QWL413	*ankr-26 (gk567) II; kyIs53 [odr-10::GFP]; EX [MKS-5::mCherry+pRF4]*
QWL414	*talp-3 (jhu511) IV; ankr-26 (gk567)II; Ex [OSM-9::GFP+MKS-5::mCherry]*

QWL415	*talp-3 (jhu511) IV; ankr-26 (gk567) II; Is [PKD-2::GFP; CC::GFP]; EX [MKS-5::mCherry+pRF4]*
QWL416	*talp-3 (jhu511) IV; ankr-26 (gk567) II; kyIs53 [odr-10::GFP]; EX [MKS-5::mCherry+pRF4]*
QWL417	*talp-3 (jhu511) IV; ankr-26 (gk567) II; EX [parl-13::TALP-3::GFP;+pRF4]*
QWL418	*talp-3 (jhu511) IV; ankr-26 (gk567) II; EX [parl-13::TALP-3Δ(425-538aa)::mCherry;+pRF4]*
QWL14	*Ex [DYF-19::GFP+MKS-5::mCherry+pRF4]*
QWL108	*talp-3 (jhu511) IV; Ex [DYF-19::GFP+MKS-5::mCherry+pRF4]*
QWL197	*ankr-26 (gk567) II; Ex [DYF-19::GFP+MKS-5::mCherry+pRF4]*
QWL218	*talp-3 (jhu511) IV; ankr-26 (gk567) II; Ex [DYF-19::GFP+MKS-5::mCherry+pRF4]*
QWL49	*Ex [ANKR-26::GFP+DYF-19::mCherry+pRF4]*
QWL178	*talp-3 (jhu511) IV; Ex [ANKR-26::GFP+DYF-19::mCherry+pRF4]*
QWL59	*dyf-19 (jhu455) V; IsOSM-6*
QWL242	*dyf-19 (jhu455) V; TALP-3 (jhu511) IV; IsOSM-6*
QWL243	*dyf-19 (jhu455) V; ankr-26 (gk567) II; IsOSM-6*
QWL295	*Ex [ANKR-26::Venus173+TALP-3::Venus155+MKS-5::mCherry+pRF4]*
QWL336	*dyf-19 (jhu455); Ex [ANKR-26::Venus173+TALP-3::Venus155+MKS-5::mCherry+pRF4]*
QWL311	*Ex [ANKR-26::Venus173+DYF-19::Venus155+MKS-5::mCherry+pRF4]*
QWL337	*TALP-3 (jhu511) IV; Ex [ANKR-26::Venus173+DYF-19::Venus155+MKS-5::mCherry+pRF4]*
QWL296	*Ex [TALP-3::Venus173+DYF-19::Venus155+MKS-5::mCherry+pRF4]*
QWL338	*ankr-26 (gk567) II; Ex [TALP-3::Venus173+DYF-19::Venus155+MKS-5::mCherry+pRF4]*
QWL189	*Ex [CHE-11::Venus173+IFT-20::Venus155+pRF4];MKS-6::mCherry*
QWL282	*talp-3 (jhu511) IV; Ex [CHE-11::Venus173+IFT-20::Venus155+pRF4];MKS-6::mCherry*
QWL291	*ankr-26 (gk567) II; Ex [CHE-11::Venus173+IFT-20::Venus155+pRF4];MKS-6::mCherry*
QWL339	*talp-3 (jhu511) IV; ankr-26 (gk567) II; Ex [CHE-11::Venus173+IFT-20::Venus155+pRF4]; MKS-6::mCherry*
QWL401	*EX [NPHP-1::GFP+TBB-4::mCherry]*
QWL402	*talp-3 (jhu511) IV; EX [NPHP-1::GFP+TBB-4::mCherry]*
QWL403	*ankr-26 (gk567) II; EX [NPHP-1::GFP+TBB-4::mCherry]*
QWL404	*talp-3 (jhu511) IV; ankr-26 (gk567) II; EX [NPHP-1::GFP+TBB-4::mCherry]*

Supplementary Table 2. Primers used in this study.

Primers for screening mutants	Sequence
talp-3(jhu511) F	GCAGATCTCAAGCCAAGTGA
talp-3(jhu511) R	CCCTACTGTAGCAGTATATGAC
talp-3(tm7883) F	CGGTTTGAGCCATTGACTTGC
talp-3(tm7883) R	CTGGCATAATGCAAACTTGAAAC
ankr-26(gk567) F	CGTGGTGGAACTTTTCAGGT
ankr-26(gk567) R	CAATTTTCACACATTCCCCC
gasr-8 (gk1232) F	CTTAGGTCACTGGTTCGAATC
gasr-8 (gk1232) R	CGAAGATTCTGGTTGGTTCG
dyf-19 (jhu455) F	CTGATTATCCGTGCGAAGTTAC
dyf-19 (jhu455) R	TCGGAGAGCCTGAAGCTTCA
hyls-1(tm3067) F	ACCCCACCATCTTGTGACTG
hyls-1(tm3067) R	AACGTTCTTCCATCGCTACT
**Primers for plasmid construction**	**Sequence**
GST-TALP-3 F	AACGGATCCATGAGTGACGAGATCCTTTCC
GST-TALP-3 R	ATAAGAATGCGGCCGCATACTTAACAACCCTATCCAAAAT
GST-TALP-3 N(1-425aa) F	AACGGATCCATGAGTGACGAGATCCTTTCC
GST-TALP-3 N(1-425aa) R	ATAAGAATGCGGCCGCAATGAAACTGAATCCAGGTGATG
GST-TALP-3 C(395-795aa) F	AACGGATCCACATCATCACTCCCACCATT
GST-TALP-3 C(395-795aa) R	ATAAGAATGCGGCCGCATACTTAACAACCCTATCCAAAAT
GST-DYF-19 F	AACGGATCCCATGTCAGACGATGAGTGGG
GST-DYF-19 R	ATAAGAATGCGGCCGCTCTCAATTATTCTGCGGCGCAACA
His-ANKR-26 F	AACGGATCCCATGAGTGGAAGACGCCAAAA
His-ANKR-26 R	ATAAGAATGCGGCCGCTCAAAACTCGGGAATTTTCTCCTGC
His-TALP-3 N(1-425aa) F	AACGGATCCATGAGTGACGAGATCCTTTCC
His-TALP-3 N(1-425aa) R	ATAAGAATGCGGCCGCATACTTAACAACCCTATCCAAAAT
VC155-TALP-3(VC155) F	AACGAATTCGGATGAGTGACGAGATCCTTTCC
VC155-TALP-3(VC155) R	AACGGTACCATACTTAACAACCCTATCCAAAAT

VN173-TALP-3(VN173)	F	AACGAATTCGGATGAGTGACGAGATCCTTTCC
VN173-TALP-3(VN173)	R	AACGGTACCATACTTAACAACCCTATCCAAAAT
VC155-ANKR-26	F	AAGCCATGGAGATGAGTGGAAGACGCCAAAA
VC155-ANKR-26	R	AACGGTACCAAACTCGGGAATTTTCTCCTGC
VC155-DYF-19	F	AACGAATTCGGATGTCAGACGATGAGTGGGG
VC155-DYF-19	R	AACGGTACCATTATTCTGCGGCGCAACAT
P*arl-13*::TALP-3Δ(425-538aa)	F	GGATTCAGTTTCTTGAAAAAGGCTGGACATTATCATGTTCCTGATT
P*arl-13*::TALP-3Δ(425-538aa)	R	CCTTTTTCAAGAAACTGAATCCAGGTGATGTCCATTGAGAATTTATG
GST-FBF1(625-1173aa)	F	AACGGATCCCTGCAGAGCCAGACCAAGCT
GST-FBF1(625-1173aa)	R	ATAAGAATGCGGCCGCGGCAGAATGGTACGCCATATT
His-ANKRD26(899-1244aa)	F	AACGGATCCGAGAATTCTCATAGTCATGAAG
His-ANKRD26(899-1244aa)	R	ATAAGAATGCGGCCGCAACCTCCAGTGAAGCCTCTG
GST-ANKRD26(685-1244aa)	F	AACGGATCCGATGACTTAACTCAGTCATCTG
GST-ANKRD26(685-1244aa)	R	ATAAGAATGCGGCCGCAACCTCCAGTGAAGCCTCTG
His-TALPID3(601-1200aa)	F	AACGAATTCAGAAGCCATTATCAAAAGCAA
His-TALPID3(601-1200aa)	R	ATAAGAATGCGGCCGCTGGCTCCGGAGGTGCAGG
His-TALPID3(399-700aa)	F	AACGAATTCACCTCACTAACTCAGCCAAA
His-TALPID3(399-700aa)	R	ATAAGAATGCGGCCGCGTCAGTTTGTGTTTTTGCTGAC
Flag-TALPID3	F	ATAAGAATGCGGCCGCAATGTTTTGGTGTGGGACTT
Flag-TALPID3	R	TGCTCTAGATTAAAACCAGTGGCATAAAC
Flag-ANKRD26	F	CGCGGATCCATGAAGAAGATTTTTAGTAAGAAGGGC
Flag-ANKRD26	R	ATAAGAAT GCGGCCGC TCAGATCATATAATTTTTCT
Flag-ANKRD26(895-1170aa)	F	CGCGGATCCATGAATTCTGAGAATTCT
Flag-ANKRD26(1097-1170aa)	F	CGCGGATCCCAAAAGGACCTAAGCCAA
Flag-ANKRD26(1147-1170aa)	F	CGCGGATCCCAACAACTGGATGATGCCCACA
HA-TALPID3	F	CGGGGTACCATGTTTTGGTGTGGGACTTGTTTTGTG
HA-TALPID3	R	ATAAGAATGCGGCCGCCTAAACCAGTGGCATAAACA
HA-ANKRD26	F	CGCGGATCCATGAAGAAGATTTTTAGTAAGAAGGGC
HA-ANKRD26	R	ATAAGAAT GCGGCCGCCCGATCATATAATTTTTCTT
