# Supplementary material for: TALPID3 and ANKRD26 selectively orchestrate FBF1 localization and cilia gating
Source: Nat Commun. 2020 May 4;11:2196. doi: 10.1038/s41467-020-16042-w (PMC7198521; doi:10.1038/s41467-020-16042-w)
Supplement: Supplementary file 3 — Reporting Summary [file 41467_2020_16042_MOESM3_ESM.pdf]

nature research

Corresponding author(s): Jinghua Hu, QIng Wei

Last updated by author(s): Mar 25, 2020

Reporting Summary

Nature Research wishes to improve the reproducibility of the work that we publish. This form provides structure for consistency and transparency in reporting. For further information on Nature Research policies, see Authors & Referees and the Editorial Policy Checklist.

Statistics

For all statistical analyses, confirm that the following items are present in the figure legend, table legend, main text, or Methods section.

n/a	Confirmed	
☐	☒	The exact sample size (*n*) for each experimental group/condition, given as a discrete number and unit of measurement
☐	☒	A statement on whether measurements were taken from distinct samples or whether the same sample was measured repeatedly
☐	☒	The statistical test(s) used AND whether they are one- or two-sided<br>*Only common tests should be described solely by name; describe more complex techniques in the Methods section.*
☒	☐	A description of all covariates tested
☒	☐	A description of any assumptions or corrections, such as tests of normality and adjustment for multiple comparisons
☐	☒	A full description of the statistical parameters including central tendency (e.g. means) or other basic estimates (e.g. regression coefficient) AND variation (e.g. standard deviation) or associated estimates of uncertainty (e.g. confidence intervals)
☒	☐	For null hypothesis testing, the test statistic (e.g. *F*, *t*, *r*) with confidence intervals, effect sizes, degrees of freedom and *P* value noted<br>*Give P values as exact values whenever suitable.*
☒	☐	For Bayesian analysis, information on the choice of priors and Markov chain Monte Carlo settings
☒	☐	For hierarchical and complex designs, identification of the appropriate level for tests and full reporting of outcomes
☒	☐	Estimates of effect sizes (e.g. Cohen's *d*, Pearson's *r*), indicating how they were calculated

Our web collection on statistics for biologists contains articles on many of the points above.

Software and code

Policy information about availability of computer code

Data collection	NIS-Elements (Nikon) was used for microscopy image acquisition.
Data analysis	Statistical analyses were performed using Excel and GraphPad Prism. Fluorescence intensities were collected using NIS-Elements (Nikon).

For manuscripts utilizing custom algorithms or software that are central to the research but not yet described in published literature, software must be made available to editors/reviewers. We strongly encourage code deposition in a community repository (e.g. GitHub). See the Nature Research guidelines for submitting code & software for further information.

Data

Policy information about availability of data

All manuscripts must include a data availability statement. This statement should provide the following information, where applicable:

- Accession codes, unique identifiers, or web links for publicly available datasets
- A list of figures that have associated raw data
- A description of any restrictions on data availability

All data supporting the findings of this study are included within the article and its Supplementary Information files or Source Data files. Materials are available from the corresponding authors upon reasonable request.

Field-specific reporting

Please select the one below that is the best fit for your research. If you are not sure, read the appropriate sections before making your selection.

☒ Life sciences ☐ Behavioural & social sciences ☐ Ecological, evolutionary & environmental sciences

Life sciences study design

All studies must disclose on these points even when the disclosure is negative.

Sample size	For percentage calculation, n=300 over 3 independent experiments. For fluorescence intensity quantification, n>30.
Data exclusions	No data were excluded.
Replication	All experiments were carried out at least three times.
Randomization	Samples were randomly assigned.
Blinding	The investigators were blinded to group allocation during data collection

Reporting for specific materials, systems and methods

We require information from authors about some types of materials, experimental systems and methods used in many studies. Here, indicate whether each material, system or method listed is relevant to your study. If you are not sure if a list item applies to your research, read the appropriate section before selecting a response.

Materials & experimental systems

n/a	Involved in the study	
☐	☒	Antibodies
☐	☒	Eukaryotic cell lines
☒	☐	Palaeontology
☐	☒	Animals and other organisms
☒	☐	Human research participants
☒	☐	Clinical data

Methods

n/a	Involved in the study	
☒	☐	ChIP-seq
☒	☐	Flow cytometry
☒	☐	MRI-based neuroimaging

Antibodies

Antibodies used	acetylated α-tubulin (T7451, diluted 1:2000 for immunofluorescence, Sigma), γ-tubulin (T6557, Sigma), FLAG (F1804, Sigma), HA (H3663, Sigma), CEP83 (HPA038161, Sigma), CEP164 (SAB3500022, Sigma), CEP290 (ab84870, Abcam), TALPID3 (24421-1-AP, Proteintech), ARL13b (17711-1-AP, Proteintech), FBF1 (11531-1-AP, Proteintech), TCTN1 (15004-1-AP, Proteintech), SCLT1 (14875-1-AP, Proteintech), IFT140 (17460-1-AP, Proteintech), beta-actin (sc-47778, diluted 1:5000 for western blotting, Santa Cruz), SMO (sc-166685, Santa Cruz), CEP89 (ab204410, Abcam), ANKRD26 (GTX128255, GeneTex), polyglutamylated tubulin (ALX-804-885-C100, diluted 1:2000 for immunofluorescence, Enzo Life Sciences), GLI-3 (AF3690, R&D Systems), ODF2 (H00004957-M01, Abnova), and Polycystin 2 (Baltimore Polycystic Kidney Disease (PKD) Research and Clinical Core Center). All antibodies were diluted 1:500 for immunofluorescence and 1:2000 for western blotting experiments unless otherwise specified.
Validation	All antibodies were described in previous publications.

Eukaryotic cell lines

Policy information about cell lines

Cell line source(s)	RPE-1 and HEK293T were obtained from American Type Culture Collection (ATCC).
Authentication	None
Mycoplasma contamination	Cell were tested for mycoplasma contamination using microscopic methods. All cell lines were mycoplasma free.
Commonly misidentified lines (See ICLAC register)	None

Animals and other organisms

Policy information about studies involving animals; ARRIVE guidelines recommended for reporting animal research

Laboratory animals	Caenorhabditis elegans of various genotypes and ages were analyzed in this manuscript. All data was collected with hermaphrodites, but PKD2 localization was collected by males.

Wild animals	Not applicable.
Field-collected samples	Not applicable.
Ethics oversight	Not applicable.

Note that full information on the approval of the study protocol must also be provided in the manuscript.
